# Progress and perspectives on BMP9-ID1 activation of HIF-1α and VEGFA to promote angiogenesis in hepatic alveolar echinococcosis

**DOI:** 10.3389/fonc.2024.1480683

**Published:** 2024-11-15

**Authors:** Fei Ke, Meng-Zhao Xu, Long Ma, Qi-Dong Chen, Bei-Bei He, Ji-De A

**Affiliations:** ^1^ The Graduate School, Qinghai University, Xining, China; ^2^ Department of Rehabilitation Medicine, Qinghai Provincial People’s Hospital, Xining, China; ^3^ General Surgery, Qinghai Provincial People's Hospital, Xining, China; ^4^ Department of Hepatic Hydatidosis, Qinghai Provincial People's Hospital, Xining, China

**Keywords:** HIF-1α, VEGFA, BMP9, ID1, angiogenesis, hepatic alveolar echinococcosis

## Abstract

Hepatic alveolar echinococcosis is a zoonotic disease with a high incidence in western China, particularly affecting plateau areas such as Qinghai, Tibet, and Xinjiang. Research has indicated the presence of neovascularization in the peripheral infiltration area of hepatic alveolar echinococcosis, with a strong correlation between angiogenesis and vascular endothelial growth factor A (VEGFA) and hypoxia-inducible factor 1-alpha (HIF-1α) overexpression. Given the similarities between hepatic alveolar echinococcosis and liver cancer, current research is focused on treating the disease by targeting related signaling pathways using molecular drugs used for liver cancer. This article aims to summarize the biological regulation of HIF-1α and VEGFA overexpression in angiogenesis related to hepatic alveolar echinococcosis, as well as the impact of the BMP9-ID1 signaling pathway on the expression levels of HIF-1α and VEGFA, providing new insights for potential treatment strategies.

## 1 Introduction

Hepatic alveolar echinococcosis (HAE) is a zoonotic parasitic disease caused by humans or animals infected with metacestode larvae of *Echinococcus multilocularis* (1). This disease is distributed globally, particularly in regions where animal husbandry is prevalent (2). The Qinghai-Tibet Plateau in China is recognized as a high-incidence area for this condition (3, 4). HAE shares biological similarities with liver cancer, both being invasive in nature, hence earning the nickname “insect cancer” (5). HAE is characterized by a prolonged incubation period and insidious onset, often resulting in a diagnosis at an advanced stage. This can subsequently lead to complications, such as cirrhosis, jaundice, and liver failure (6). The radical cure rate of HAE is notably low and poses a significant threat to the health and safety of individuals (7). Neovascularization has been found to form in the infiltration area around the HAE lesion, and -inducible factor 1-alpha (HIF-1α), vascular endothelial growth factor A (VEGFA), and vascular endothelial growth factor receptor (VEGFR) signaling pathways may be involved in this process. Because the biological characteristics of liver cancer are similar to those of HAE, the potential for applying molecular-targeted drugs for liver cancer treatment to HAE has garnered significant research attention. This has gradually become the focus of current research. Research on the liver cancer signaling pathway has revealed that bone morphogenetic protein 9 (BMP9) and inhibitor of DNA binding 1 (ID1) can activate the expression of HIF-1α and VEGFA, which affects the angiogenesis of liver cancer lesions (8). In healthy cases, BMP9 has antagonistic proliferative and stabilizing effects on mature and healthy blood vessels, but overexpressed BMP9 protein can promote the angiogenesis of hepatocellular carcinoma (9). ID1 plays a negative regulatory role in normal conditions, inhibits cell differentiation, promotes tumor occurrence and angiogenesis. BMP9 and ID1 have the effect of promoting angiogenesis in liver lesions, both of which may be highly expressed in HAE lesions, and affect the expression levels of HIF-1α, VEGFA and VEGFR. This process induces angiogenesis around the HAE lesion. This review summarizes the distribution characteristics of microvessel density (MVD) in HAE lesions at this stage, the biological regulation of HIF1 a and VEGFA overexpression in angiogenesis in HAE, and the related research progress of the effect of BMP9-ID1 signaling pathway on HIF-1α and VEGFA expression with the aim of elucidating new directions for molecular biology research and molecular targeted therapies for HAE (**Appendix Table 1**).

## 2 Distribution characteristics of MVD in HAE lesions

### 2.1 Biological characteristics of HAE lesions

The pathologic structure of HAE is a myriad collection of small vesicles, with a large body view of a single giant mass type, hard and poorly demarcated from the surrounding tissue. He proliferates by way of outgrowth or infiltration, constantly producing new vesicles that penetrate deep into the tissue. It can not only directly invade neighboring tissues and structures, but also metastasize to the retroperitoneum and distant organs, such as the brain and lungs, through lymphatic and blood transport (10). It can be seen that the invasive mode of HAE is similar to that of Hepatic Cell Carcinoma(HCC) which has the characteristic of metastasis to adjacent tissues and organs, even to distant organs. Both HAE and HCC show dense occupying foci in the unhepatic liver on imaging, so it is difficult to differentiate between them by simple imaging. However, due to the blood supply of HAE lesions, their centers often show irregular necrotic liquefied cavities and scattered or irregular flaky calcified foci, while HCC often has a rich blood supply in its center in order to maintain its persistent invasive properties, so the two are distinguished from each other by various angiographic imaging tests. In addition, immunological testing is an important auxiliary method for the diagnosis and differential diagnosis of HAE, and the immunopurified natural antigen 2β-methoxy-2-deethoxyphantomolin (Em2) has good sensitivity (89.3%) and specificity (98%) for vesicular schistosomiasis, whereas HCC often causes an increase in alphafetoprotein (AFP) (10, 11) (**Appendix Table 1**).

Measuring MVD in tumor tissue to reflect the degree of tumor vascularization has become a reliable quantitative index for evaluating tumor invasion and growth (12). Abundant blood supply promotes malignant reproduction of tumor cells. HAE has similar biological characteristics to malignant tumors in terms of invasive growth and vascular invasion, and the invasion characteristics of HAE are highly correlated with the activity of echinococcosis around the lesion, which is called the “peripheral zone.” New blood vessels provide a microenvironment for the growth and proliferation of worms and maintain echinococcotic activity (13, 14). The use of MVD as a reference for the degree of infiltration of HAE lesions may allow precise assessment of the vascular supply of lesions and provide a new means for individualized treatment plans to improve long-term prognosis.

### 2.2 Imaging features of HAE lesions

Based on the differences in blood supply distribution in HAE and using MVD as a reference, special imaging methods can indirectly reflect the distribution of blood supply in HAE lesions as internal (“lack of blood supply”) or peripheral (“rich blood supply”) infiltration areas (15). With the continuous application of advanced technologies such as color Doppler ultrasonography, computed tomography(CT) perfusion imaging, positron emission computed tomography, and magnetic resonance imaging for the diagnosis and differential diagnosis of HAE, the study of microvascular density in HAE lesions is also undergoing major developments (16). In recent years, to verify the distribution characteristics of blood supply in HAE lesions, Song T et al. (16, 17) used ultrasonography in combination with MVD and found that there was a rich vascular supply in the infiltrating and proliferating areas of HAE through correlation studies in animal experiments, which revealed the pathologic characteristics of HAE; Yao B et al. (18, 19) demonstrated that the peripheral infiltration of human liver vesicular echinococcus granulose tissue carries abundant blood flow signals by CT perfusion imaging; Zheng J, Jiang Y, and other scholars evaluated the distribution of blood supply in HAE by using dual-source and dual-energy CT, concluded that the degree of lesion enhancement was positively correlated by MVD, and the highest counts of MVD were found in the marginal zone of HAE lesions, which further interpreted the distribution characteristics of the blood supply of HAE lesions (20, 21). Although using imaging techniques to study the microvascular density of HAE lesions can only abstractly explain the “lack of blood supply” and “rich blood supply” distribution characteristics in HAE lesions, it also provides a basis for studying angiogenesis in the infiltrating zones of HAE lesions (16–21) (**Appendix Table 1**).

### 2.3 Proliferation of HAE lesions in relation to angiogenic factors

Imaging and other methods have indicated that the “blood-rich” character of the periphery of the infiltrated area of the HAE lesion may be related to the neovascularization around the lesion area. At the molecular biological level, there is a lack of evidence as to whether this peripheral “blood-rich” character of HAE lesions is related to neoangiogenesis. The nutritional supply of hepatic malignant tumors is supported by neovascularization, and studies have demonstrated that neovascularization is highly correlated with the increased secretion of factors such as vascular endothelial growth factor (VEGF) (22, 23). Angiogenesis-related signaling pathways have become the main molecular basis of hepatocarcinogenesis. Based on the signaling pathway studies of hepatic malignant tumors, the study of the signaling pathway of peripheral vascular neovascularization in HAE lesions has become a major putative line in recent years. Some studies have demonstrated the existence of neovascularization in the periphery of HAE, and it is suspected that there is a correlation with the expression of factors, such as VEGF and CD34 (24) (**Appendix Table 1**).

## 3 Relationship between the HIF-1α/VEGFA/VEGFR pathway and the proliferation of HAE lesions

### 3.1 Overview of the HIF-1α/VEGFA/VEGFR pathway

Hypoxia inducible factor-1 (HIF-1) is a heterodimer composed of two subunits, α and β, in which HIF-1α is the main component of regulating hypoxia signal (25). Under hypoxia, HIF-1α binds to HIF-1β to form an active HIF complex capable of initiating transcription, which binds to the hypoxia-responsive element (HRE) of hypoxia-responsive genes (e.g., VEGF, erythropoietin, etc.) to recruit other transcription factors, triggering a series of hypoxia-tolerant adaptive responses (26). The increased expression of HIF-1α can regulate many downstream target genes. Target genes regulated by HIF-1α play important roles in various aspects of tumor cell energy metabolism, apoptosis, angiogenesis, proliferation, and metastasis (27). The rapid proliferation of malignant tumors places them in a long-term hypoxic state. To adapt to the hypoxic environment, HIF-1α in human tissues will be overexpressed to stimulate the downstream targets and participate in the expression of tumor genes related to translation modification. To meet its own energy metabolism requirements, the tumor tissue promotes the formation of a large number of new blood vessels at the lesion. Under hypoxic conditions, HIF-1α will be overexpressed to participate in the proliferation, budding, and migration of neovascular endothelial cells (28).

Vascular endothelial growth factor A (VEGFA) is a dimeric glycoprotein that belongs to the VEGF family, which includes VEGFB, VEGFC, VEGFD, and placental growth factor (PIGF). VEGFA is the most effective vascular permeability mechanism inducer in the VEGF family. VEGFA enhances microvascular permeability and induces angiogenesis by upregulating urokinase-type plasminogen activator(uPA), tissue plasminogen activator (tPA), and plasminogen activator inhibitor 1(PAI-1) expression (29). However, the formation of new blood vessels is complex. VEGFA induction must be mediated by receptors on the target cells. Presently, the confirmed VEGF family of receptors includes VEGFR1, VEGFR2, and VEGFR3. VEGFR1 and VEGFR2 are primarily distributed in vascular endothelial cells (30). VEGFR3 is often expressed in the lymphatic endothelial cells. There is a complete MAP kinase-related pathway involved in the signal transduction of VEGFR2, which can promote chemotactic mitosis, thus playing a role in mediating the formation of new blood (31). The stimulatory and inhibitory effects of VEGFR1 on the VEGFR2 pathway remain controversial. In knockout experiments of related animal models, a mouse model lacking the VEGFR1 tyrosine kinase region and transmembrane region died due to vascular malformations. During embryogenesis, VEGFR1 induces the binding of VEGFA to VEGFR2 to promote angiogenesis (32). Studies have also shown that VEGFR1 inhibits endothelial cell proliferation via the VEGFR2 signaling pathway (33). Although the stimulatory and inhibitory effects of VEGFR1 are controversial, the phosphorylation of VEGFR2 after VEGFA stimulation was observed on all intact cell surfaces. This signaling pathway has been shown to mediate neovascularization in a large number of studies (29–33) (**Appendix Table 1**).

**Figure 1:**
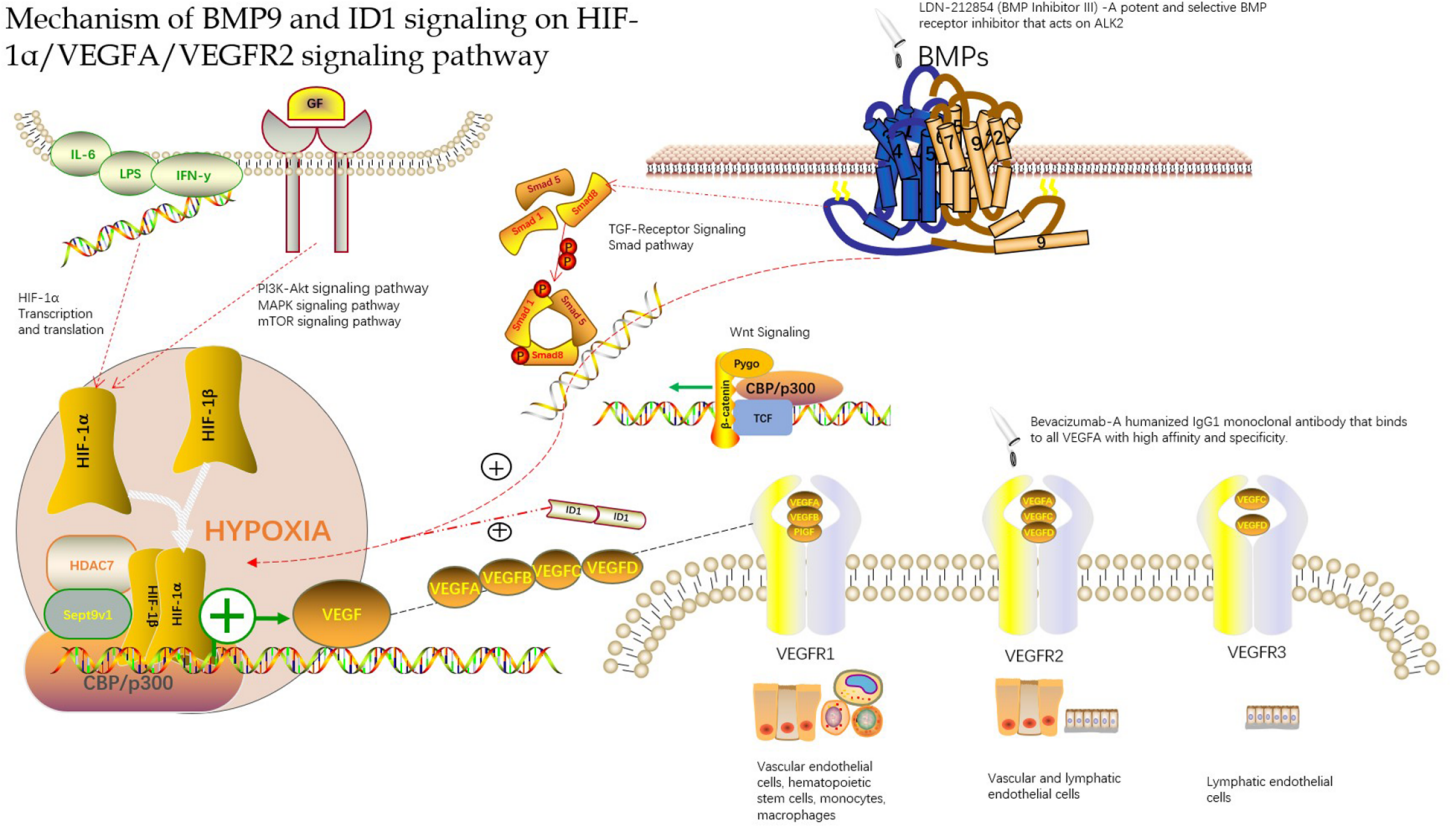
Mechanism of BMP9 and ID1 signaling on HIF-1α/VEGFA/VEGFR2 signaling pathway.

In a hypoxic environment, the expression of VEGFA and VEGFR in the tissue is greatly increased, which may be related to the continuous expression of hypoxia-inducible factors in the hypoxic microenvironment (34). HIFs can be used as upstream signaling factors to bind to the initiation regions of VEGFA and VEGFR and increase their expression. Wang W (35) found that there was a significant positive correlation between MVD and HIF-1α and VEGF, as well as between HIF-1α and VEGF levels by establishing an animal model of hepatocellular carcinoma, which further proved that HIF-1α and VEGF play an important role in the occurrence of hepatocellular carcinoma and participate in the formation of new blood vessels in hepatocellular carcinoma. The HIF-1α, VEGF and VEGFR signaling pathways may also be involved in the formation of new blood vessels in the peripheral infiltration area of HAE, which has similar biological characteristics to those of hepatocellular carcinoma (**Figure 1**).

### 3.2 Expression of HIF-1α, VEGFA, and VEGFR in the infiltration zone (surrounding tissue) of HAE lesions

At present, with the deepening of HAE genomics research, many scientists have found that the lesion and its peripheral infiltration zone has rich microvascular density, and it has been proved by experiments that HIF-1α, VEGFA, and VEGFR signaling pathway may be involved in the formation of its microvessels and participate in the occurrence and development of HAE (36–39). Zhang et al. (38) found that MVD and VEGF in the lesion tissues of gerbils infected with alveolar echinococcosis were highly expressed and were higher than those in the surrounding liver tissues by measuring MVD and vascular endothelial growth factor in the liver alveolar echinococcosis tissues of gerbils. The expression levels of MVD and VEGF in the liver tissues around the lesions increased progressively with prolonged onset time. The animal model test showed that there was new angiogenesis in the liver alveolar echinococcosis lesions and surrounding liver tissues, which was likely related to VEGF and other related signaling factors. Jiang HJ (36) studied the role of VEGFA/VEGFR2 in the angiogenesis of HAE in mice. The results showed that VEGFA and VEGFR2 were mainly expressed in the fibrous endothelial cell infiltration area between the outer cyst wall and the liver cells and the inner cyst wall of multilocular echinococcosis, which also indicate that neovascularization may occur in these two areas. Immunohistochemistry and enzyme-linked immunosorbent assay results showed that VEGFA plays an important role in early and middle angiogenesis in *Echinococcus multilocularis* (39). In animal studies, it has been found that angiogenesis-related factors are highly expressed in HAE models, and the expression of VEGFA is different in the lesions and surrounding liver tissues; however, there are some differences in the expression levels of lesions and surrounding liver tissues. Wang J et al. (37) found that the expression of VEGF did not increase in 27 cases of human hepatic alveolar echinococcosis by immunohistochemical analysis of VEGF in 27 cases of human hepatic alveolar echinococcosis, which is different from animal experiments. This may be due to the incubation period of patients from infection to disease onset, which usually takes years or even longer. We cannot exclude the possibility that the expression of VEGF and other related factors increase with disease progression.

In conclusion, many studies have found that HAE lesions and peripheral infiltration exhibit neovascularization and that MVD and VEGF are highly expressed. Although there are some differences in the expression of these indicators of angiogenesis in HAE lesions and peripheral infiltration of the liver tissue, these findings provide a new direction for the application of VEGF signaling pathway-targeted drugs in the treatment of HAE to a certain extent. Sang et al. (40) found that bevacizumab inhibited the effect of VEGF and the formation of blood vessels in the treatment of hepatic alveolar echinococcosis in rats via hepatic artery infusion (**Figure 1**). There is still a blind spot in the understanding of the role of HIF-1α, VEGF, and VEGFR signaling pathways in HAE, which requires further in-depth research, which may provide a new way for HAE vascularization research and anti-vascular treatment.

## 4 Regulation of HIF-1α/VEGFA pathway expression by BMP9, ID1

### 4.1 Overview of BMP9, ID1

Bone morphogenetic proteins (BMPs) are members of the transforming growth factor beta(TGF-β) superfamily, which play a role in regulating cell growth and differentiation during embryonic development (41). Bone morphogenetic protein 9(BMP9) is a member of the BMPs family that is mainly secreted by hepatic stellate cells and participates in a variety of biological processes, including angiogenesis, iron balance, and energy metabolism.

In the field of liver research, BMP9 can maintain the polarization and function of hepatocytes under healthy conditions and has an antagonistic proliferation and stability effect on mature and healthy blood vessels. However, BMP9 overexpression promotes angiogenesis of hepatocellular carcinoma. Studies have shown that BMP9 is overexpressed in approximately 40% of patients with hepatocellular carcinoma (42). Herrera et al. (43) studied the effect of BMP9 on HCC cell line markers and cell migration by measuring liver specimens and found that BMP9 could induce epithelial-mesenchymal transition(EMT) in HCC cell lines by activating the smad1 pathway, making it a potential promoter sequence of HCC. Researchers have also found that BMP9 stimulation of liver cancer cells can trigger the phosphorylation of Smad1/5/8 and upregulation of inhibitor of differentiation-1 protein(ID1). This study also showed that BMP9 not only promotes the proliferation response but also plays an anti-apoptotic role through chemical inhibitors, ligand trapping, and gene silencing (44). BMP9 exhibits distinct functions under disease and healthy conditions. In disease conditions, it not only promotes the proliferation and survival of liver cancer cells but also induces the transformation of epithelial cells into mesenchymal cells, thereby promoting tumor progression.

Inhibitor of differentiation-1 protein (ID1) is a DNA-binding inhibitor. ID1 is a member of the ID family, which plays a negative regulatory role and inhibits cell differentiation (45). The ID1 protein is rarely expressed in healthy humans, but a large number of ID1 proteins with different characteristics can be found in tumors. ID1 has been confirmed to inhibit cell differentiation, promote cell proliferation, and promote tumorigenesis and angiogenesis (46). In particular, it promotes tumorigenesis and angiogenesis, thereby providing a new target for tumor treatment. Several studies have shown that ID1 is highly expressed in most tumor tissues and is positively correlated with tumor invasion and metastasis (47–49). Han et al. (47) detected the expression of ID1 in gastric cancer tissues by immunohistochemistry and western blotting. The expression level of ID1 protein in gastric cancer tissues was significantly higher than that in surrounding tissues. ID1 expression positively correlated with the degree of differentiation, invasion, and metastasis of gastric cancer. The level of the ID1 protein in gastric cancer tissues with a low degree of differentiation was higher than that in gastric cancer tissues with a high degree of differentiation, indicating that the expression of ID1 may be related to the occurrence and development of gastric cancer. Similarly, in the field of liver cancer, Matsuda Y et al. (49) showed that increased ID1 expression may be involved in the early stages of liver cancer. At the same time, for the role of ID1 in tumor angiogenesis, Ciarrocchi et al. (48) found that the lack of ID1 in the bone marrow destroyed tumor angiogenesis, thereby inhibiting tumor invasion and development, which indirectly verified that ID1 may be involved in tumor angiogenesis.

BMP9 and ID1 play important roles in the occurrence, development, and invasion of malignant tumors. Studies have shown that BMP9 and ID1 are overexpressed in malignant liver tumor tissues and play important roles in the angiogenesis of liver cancer tissues (8, 9, 50). Although the specific regulatory mechanism of the two is not clear, these findings undoubtedly provide a new key for targeted therapy of liver cancer and lead to new thinking about whether the two may also be involved in the development of HAE.

### 4.2 BMP9-ID1 can activate HIF-1α and VEGFA expression

Angiogenesis is considered to be a key player in the development of hepatocellular carcinoma. VEGFA is the primary stimulator of angiogenesis, and VEGFA is the direct target of HIF-1α. Therefore, targeting VEGFA and its upstream proteins has constituted a promising new strategy for antiangiogenic therapy. Chen et al. (8, 50) found that BMP9 is a key factor in the induction of hepatocellular carcinoma in a study of the hepatocellular carcinoma signaling pathway. They found that BMP9-ID1 signaling promotes epithelial cell adhesion molecule(EpCAM)-positive cancer stem cells by activating Wnt/β-catenin signaling, which indicates that BMP9 and ID1 may be involved in the occurrence and development of hepatocellular carcinoma. Based on this, they further studied the functional role of BMP9 signaling in HCC angiogenesis. They first explored the correlation between BMP9 and angiogenesis proteins VEGFR2 and HIF-1α in the cancer genome atlas (HCC) cohort and demonstrated that BMP9 is involved in HCC angiogenesis. They further studied the mechanism of BMP9-induced HIF-1α/VEGFA expression and revealed that VEGFA was abundantly expressed in ID1-expressing HCC cells. To confirm whether ID1 is involved in the regulation of this signal transduction, they proved this conjecture by ID1 silencing/overexpression experiments and found that ID1 enhanced the stability of HIF-1α protein in HCC cells. After, they hypothesized that ID1 can induce the expression of HIF-1α and VEGFA by regulating BMP9. Immunohistochemistry and protein determination by knocking down ID1 protein revealed that ID1-knockdown reduced BMP9-induced HIF-1α/VEGFA expression and secretion. Overexpression of ID1 increased the expression and secretion of HIF-1α/VEGFA protein and promoted the formation of new blood vessel lumens. The discovery that BMP9-ID1 can activate the expression of HIF-1α and VEGFA supports a new strategy for developing targeted therapies for hepatocellular carcinoma. Although the mechanism by which angiogenesis is promoted requires further research, it is a new breakthrough in understanding the regulation axis of the HIF-1α/VEGFA/VEGFR signaling pathway, thus supporting the targeted treatment of hepatocellular carcinoma and of HAE owing to its similarity with hepatocytes (**Figure 1**) (**Appendix Table 1**).

## 5 Summary and outlook

HAE is closely associated with angiogenesis. Through the determination of MVD, it was found that the blood supply of HAE lesions was characterized by an lack of internal blood supply and a peripheral infiltration area rich in blood supply. Through a large number of animal experiments and the study of the protein expression of angiogenesis factors in human liver tissue, angiogenesis-related factors were found to be highly expressed in the peripheral infiltration area of HAE lesions; thus, angiogenesis in the peripheral infiltration area of HAE lesions may be highly correlated with the conduction of HIF-1α/VEGFA/VEGFR signaling pathway. Due to the similar biological characteristics between HAE and hepatocellular carcinoma, the possibility of applying drugs targeting the angiogenesis signaling pathway of hepatocellular carcinoma for the treatment of HAE has garnered significant research attention. Some studies have found that BMP9-ID1 can activate the expression of HIF-1α and VEGFA in the blood supply of liver cancer, which provides a new strategy for targeted liver cancer therapies. Similarly, it is possible to use drugs that inhibit BMP9 and ID1 expression to treat HAE. There remains a lack of evidence demonstrating that BMP9-ID1 activates the expression of HIF-1α and VEGFA, and there are numerous problems hindering the study of the signaling pathways involved in HAE neovascularization. Targeted therapies for the treatment of HAE are expected to be researched and developed further.

## Appendix

**Table 1:** List of acronyms and some of the antagonist targets.

HAE	Hepatic Alveolar Echinococcosis
HCC	Hepatic Cell Carcinoma
HIF-1	Hypoxia Inducible Factor-1
HIF-1α	Hypoxia-inducible Factor 1-alpha
HIF-1β	Aryl hydrocarbon Receptor Nuclear Translocator, ARNT1
VEGF	Vascular Endothelial Growth Factor
VEGFA	Vascular Endothelial Growth Factor-A
VEGFB	Vascular Endothelial Growth Factor-B
VEGFC	Vascular Endothelial Growth Factor-C
VEGFD	Vascular Endothelial Growth Factor-D
PIGF	Placental Growth Factor
VEGFR	Vascular Endothelial Growth Factor Receptor
VEGFR1	Vascular Endothelial Growth Factor Receptor 1
VEGFR2	Vascular Endothelial Growth Factor Receptor 2
VEGFR3	Vascular Endothelial Growth Factor Receptor 3
BMPs	Bone Morphogenetic ProteinS
TGF-β	Transforming Growth Factor Beta
BMP9	Bone Morphogenetic Protein 9
ID1	Inhibitor Of DNA Binding 1
MVD	Micro Vessel Density
CD34	CD34 Molecule
CT	Computed Tomography
AFP	Alphafetoprotein
Em2	A monomeric sesquiterpene lactone called 2β-methoxy-2-deethoxyphantomolin
HRE	Hypoxia-responsive Element
uPA	Urokinase-type Plasminogen Activator
tPA	Tissue Plasminogen Activator
PAI-1	Plasminogen Activator Inhibitor 1
EMT	Epithelial-mesenchymal Transition
EpCAM	Epithelial Cell Adhesion Molecule
Bevacizumab	A humanized IgG1 monoclonal antibody that binds to all VEGFA with high affinity and specificity
LDN-212854	BMP Inhibitor III -A potent and selective BMP receptor inhibitor that acts on ALK2

## References

[1] Wen H Vuitton L Tuxun T Li J Vuitton DA Zhang W. Echinococcosis: advances in the 21st century. Clin Microbiol Rev. (2019) 32:e00075-18. doi: 10.1128/CMR.00075-18 30760475 PMC6431127

[2] Fu MH Wang X Han S Guan YY Bergquist R Wu WP. Advances in research on echinococcoses epidemiology in China. Acta Trop. (2021) 219:105921. doi: 10.1016/j.actatropica.2021.105921 33878307

[3] Dang Z Fu Y Duo H Fan H Qiao Z Guo Z. An epidemiological survey of echinococcosis in intermediate and definitive hosts in qinghai province, China. Trop BioMed. (2017) 34:483–90. 33593033

[4] Wang LY Qin M Liu ZH Wu WP Xiao N Zhou XN. Prevalence and spatial distribution characteristics of human echinococcosis in China. PloS Negl Trop Dis. (2021) 15:e0009996. doi: 10.1371/journal.pntd.0009996 34962928 PMC8789093

[5] Zhu YY Wu WP. Advances in echinococcosis prevention and control programs and research in China and elsewhere around the world. J Parasitic Biol. (2016) 11:284–86. doi: 10.13350/j.cjpb.160321

[6] Deng X Yang-Dan CR Zhang LQ Wang ZX Wang JJ Wang KQ. Current status of the surgical treatment of hepatic alveolar echinococcosis. J Clin Hepatol. (2021) 37:1963–65. doi: 10.3969/j.issn.1001-5256.2021.08.047

[7] Brunetti E Kern P Vuitton DA. Expert consensus for the diagnosis and treatment of cystic and alveolar echinococcosis in humans. Acta Trop. (2010) 114:1–16. doi: 10.1016/j.actatropica.2009.11.001 19931502

[8] Chen H Nio K Tang H Yamashita T Okada H Li Y. BMP9-ID1 signaling activates HIF-1α and VEGFA expression to promote tumor angiogenesis in hepatocellular carcinoma. Int J Mol Sci. (2022) 23:1475. doi: 10.3390/ijms23031475 35163396 PMC8835914

[9] Scharpfenecker M van Dinther M Liu Z van Bezooijen RL Zhao Q Pukac L. BMP-9 signals via ALK1 and inhibits bFGF-induced endothelial cell proliferation and VEGF-stimulated angiogenesis. J Cell Sci. (2007) 120:964–72. doi: 10.1242/jcs.002949 17311849

[10] Chinese Doctor Association, Chinese College of Surgeons (CCS) Chinese Committee for Hadytidology (CCH). Expert consensus on diagnosis and treatment of hepatic cystic and alveolar echinococcosis (2019 edition). Chin J Dig Surg. (2019) 18:711–21. doi: 10.3760/cma.j.issn.1673-9752.2019.08.002

[11] Department of Medical Administration National Health Commission of the People′s Republic of China. Guideline for diagnosis and treatment of primary liver cancer (2024 edition). J Hepatopancreatobiliary Surg. (2024) 23:429–78. doi: 10.3760/cma.j.cn115610-20240415-00203

[12] Weidner N. Tumour vascularity and proliferation: clear evidence of a close relationship. J Pathol. (1999) 189:297–99. doi: 10.1002/(SICI)1096-9896(199911)189:3<297::AID-PATH434>3.0.CO;2-O 10547589

[13] Ren B Wang J Miao Z Xia Y Liu W Zhang T. Hepatic alveolar echinococcosis: predictive biological activity based on radiomics of MRI. BioMed Res Int. (2021) 2021:6681092. doi: 10.1155/2021/6681092 33997041 PMC8108638

[14] Chen L. Consistency study of the activity in the infiltrative belt surrounding hepatic alveolar echinococcosis in rats by gray-scale contrast-enhanced ultrasonography. Urumqi City, China: Xin Jiang Medical University (2019). [master’s thesis].

[15] JD A Chai JP Zhao SY An XR Yang JY An X. Research progress on infiltrating zone and microvascular invasion of hepatic alveolar echinococcosis. Zhonghua Yu Fang Yi Xue Za Zhi. (2022) 56:1514–19. doi: 10.3760/cma.j.cn112150-20211118-01062 36274623

[16] Song T Li HT Yang LF Yao LH Wen H. Contrast-enhanced ultrasonography of hepatic alveolar echinococcosis in rats: the correaltion of imaging fratures and histologic microvascular density. Zhongguo Ji Sheng Chong Xue Yu Ji Sheng Chong Bing Za Zhi. (2014) 32:200–04. 25223055

[17] Song T Lv YQ Yao LH Zhao Q Yang L. Effects of contrast-enhanced grey scale ultrasonography on characterization of hepatic alveolar echinococcosis. Chin J Ultrasonogr. (2008) 17:133–35. doi: 10.3321/j.issn:1004-4477.2008.02.012

[18] Yao B Wang HT Liu WY Wang J Jiang Y Wang J. Peripheral zone of hepatic alveolar echinococcosis: CT perfusion and pathology. Chin Computed Med Imaging. (2010) 16:215–20. doi: 10.19627/j.cnki.cn31-1700/th.2010.03.011

[19] Yao B. Peripheral Zone of Hepatic Alveolar Echinococcosis: CT Perfusion and Pathology. Xin Jiang Medical University (2010). [master’s thesis].

[20] Zheng J Wang J Zhao J Meng X. Diffusion-weighted MRI for the initial viability evaluation of parasites in hepatic alveolar echinococcosis: comparison with positron emission tomography. Korean J Radiol. (2018) 19:40–6. doi: 10.3348/kjr.2018.19.1.40 PMC5768505 29353998

[21] Jiang Y Wang J Xiao H Li TT Liu H Liu WY. Assessment of vascularity in liver alveolar echinococcosis with dual energy CT:a correlation between iodine quantification and histopathologic parameters. J Xinjiang Med Univ. (2015) 38:1207–12. doi: 10.3969/j.issn.1009-5551.2015.10.002

[22] Campagnolo L Telesca C Massimiani M Stuhlmann H Angelico M Lenci I. Different expression of VEGF and EGFL7 in human hepatocellular carcinoma. Dig Liver Dis. (2016) 48:76–80. doi: 10.1016/j.dld.2015.09.019 26542361

[23] Morse MA Sun W Kim R He AR Abada PB Mynderse M. The role of angiogenesis in hepatocellular carcinoma. Clin Cancer Res. (2019) 25:912–20. doi: 10.1158/1078-0432.CCR-18-1254 30274981

[24] Li T Zhang SJ. A review of vascular regeneration in the invasive growth process of Echinococcus multilocularis. Chin J Zoonoses. (2014) 30:1071–74. doi: 10.3969/cjz.j.issn.1002-2694.2014.10.018

[25] Semenza GL. Hypoxia-inducible factor 1 (HIF-1) pathway. Sci STKE. (2007) 2007:cm8. doi: 10.1126/stke.4072007cm8 17925579

[26] Semenza GL. HIF-1 and mechanisms of hypoxia sensing. Curr Opin Cell Biol. (2001) 13:167–71. doi: 10.1016/s0955-0674(00)00194-0 11248550

[27] Li YM Chen YQ. Hypoxia-inducible factor-1, a novel target for cancer cell apoptosis and angiogenesis. Chin J Cancer Prev Treat. (2008) 12):948–50. doi: 10.16073/j.cnki.cjcpt.2008.12.020

[28] Krock BL Skuli N Simon MC. Hypoxia-induced angiogenesis: good and evil. Genes Cancer. (2011) 2:1117–33. doi: 10.1177/1947601911423654 PMC3411127 22866203

[29] Melincovici CS Bosca AB Susman S Marginean M Mihu C Istrate M. Vascular endothelial growth factor (VEGF) - key factor in normal and pathological angiogenesis. Rom J Morphol Embryol. (2018) 59:455–67. 30173249

[30] Perez-Gutierrez L Ferrara N. Biology and therapeutic targeting of vascular endothelial growth factor a. Nat Rev Mol Cell Biol. (2023) 24:816–34. doi: 10.1038/s41580-023-00631-w 37491579

[31] Wagner MJ Lyons YA Siedel JH Dood R Nagaraja AS Haemmerle M. Combined VEGFR and MAPK pathway inhibition in angiosarcoma. Sci Rep. (2021) 11:9362. doi: 10.1038/s41598-021-88703-9 33931674 PMC8087824

[32] Hiratsuka S Nakao K Nakamura K Katsuki M Maru Y Shibuya M. Membrane fixation of vascular endothelial growth factor receptor 1 ligand-binding domain is important for vasculogenesis and angiogenesis in mice. Mol Cell Biol. (2005) 25:346–54. doi: 10.1128/MCB.25.1.346-354.2005 PMC538773 15601855

[33] Rahimi N Dayanir V Lashkari K. Receptor chimeras indicate that the vascular endothelial growth factor receptor-1 (VEGFR1) modulates mitogenic activity of VEGFR2 in endothelial cells. J Biol Chem. (2000) 275:16986–92. doi: 10.1074/jbc.M000528200 10747927

[34] Yu DC Chen J Ding YT. Hypoxic and highly angiogenic non-tumor tissues surrounding hepatocellular carcinoma: the ‘niche’ of endothelial progenitor cells. Int J Mol Sci. (2010) 11:2901–09. doi: 10.3390/ijms11082901 PMC2996747 21152281

[35] Wang W Xu GL Jia WD Wang ZH Li JS Ma JL. Expression and correlation of hypoxia-inducible factor-1alpha, vascular endothelial growth factor and microvessel density in experimental rat hepatocarcinogenesis. J Int Med Res. (2009) 37:417–25. doi: 10.1177/147323000903700217 19383236

[36] Zhang SJ Wu HX Li Q Wu XW Zhang XZ Wang YZ. Expressions of MVD-CD34 and VEGF in hepatic alveolar hydatid tissue in gerbil model and their clinical significances. Chin J Bases Clinics Gen Surg. (2020) 38:673–81. doi: 10.12140/j.issn.1000-7423.2020.06.001

[37] Jiang HJ Gui XW Guo LJ Yang XF Wang XY Chen XL. Expression and angiogenic effect of VEGFA/VEGFR2 in mice hepatic metacestode tissue of Echinococcus multilocularis. Chin J Parasitol Parasitic Dis. (2011) 45:1036–39. doi: 10.3760/cma.j.issn.1005-1201.2011.11.010

[38] Zhou Q Yang XF Han HH Guo LJ Jiang HJ Wang XY. Correlation between angiogenesis and disease progression of hepatic Echinococcus multilocularis in C57BL/6 mice. Chin J Parasitol Parasitic Dis. (2015) 22:134–38. doi: 10.7507/1007-9424.20150038

[39] Wang J Ren B Liu W Wen H Qing S Xie WD. The correlation of CT perfusion imaging with microvessel density and vascular endothelial growth factor in hepatic alveolar echinococcosis. Chin J Radiol. (2019) 37:302–10. doi: 10.12140/j.issn.1000-7423.2019.03.011

[40] Sang ZJ Zhu DW Ji WZ Gu JP Zhang HX Ren WX. Dynamic observation on the serum level of vascular endothelial growth factor in experimental rats with hepatic alveolar echinococcosis after hepatic arterial infusion of bevacizumab treatment. J Interventional Radiol. (2014) 23:516–19. doi: 10.3969/j.issn.1008-794X.2014.06.014

[41] Fabregat I Herrera B Sanchez A. Editorial special issue TGF-beta/BMP signaling pathway. Cells. (2020) 9(11):2363. doi: 10.3390/cells9112363 33121103 PMC7693659

[42] Jiang QQ Liu BB Xu KS. New insights into BMP9 signaling in liver diseases. Mol Cell Biochem. (2021) 476:3591–600. doi: 10.1007/s11010-021-04182-6 34019202

[43] Herrera B Dooley S Breitkopf-Heinlein K. Potential roles of bone morphogenetic protein (BMP)-9 in human liver diseases. Int J Mol Sci. (2014) 15:5199–220. doi: 10.3390/ijms15045199 PMC4013558 24670474

[44] Herrera B Garcia-Alvaro M Cruz S Walsh P Fernandez M Roncero C. BMP9 is a proliferative and survival factor for human hepatocellular carcinoma cells. PloS One. (2013) 8:e69535. doi: 10.1371/journal.pone.0069535 23936038 PMC3720667

[45] Yokota Y Mori S. Role of id family proteins in growth control. J Cell Physiol. (2002) 190:21–8. doi: 10.1002/jcp.10042 11807807

[46] Zhao Z Bo Z Gong W Guo Y. Inhibitor of differentiation 1 (id1) in cancer and cancer therapy. Int J Med Sci. (2020) 17:995–1005. doi: 10.7150/ijms.42805 32410828 PMC7211148

[47] Han S Gou C Hong L Liu J ZheyiHan Liu C. Expression and significances of id1 helix-loop-helix protein overexpression in gastric cancer. Cancer Lett. (2004) 216:63–71. doi: 10.1016/j.canlet.2004.07.035 15575081

[48] Ciarrocchi A Jankovic V Shaked Y Nolan DJ Mittal V Kerbel RS. Id1 restrains p21 expression to control endothelial progenitor cell formation. PloS One. (2007) 2:e1338. doi: 10.1371/journal.pone.0001338 18092003 PMC2129121

[49] Matsuda Y Yamagiwa S Takamura M Honda Y Ishimoto Y Ichida T. Overexpressed id-1 is associated with a high risk of hepatocellular carcinoma development in patients with cirrhosis without transcriptional repression of p16. Cancer. (2005) 104:1037–44. doi: 10.1002/cncr.21259 15999366

[50] Chen H Nio K Yamashita T Okada H Li R Suda T. BMP9-ID1 signaling promotes EpCAM-positive cancer stem cell properties in hepatocellular carcinoma. Mol Oncol. (2021) 15:2203–18. doi: 10.1002/1878-0261.12963 PMC8333780 33834612

